# Genetic and Pharmacological Inhibition of NOX4 Protects Against Rhabdomyolysis-Induced Acute Kidney Injury Through Suppression of Endoplasmic Reticulum Stress

**DOI:** 10.3390/antiox14101162

**Published:** 2025-09-25

**Authors:** Zhuyun Zhang, Jiameng Li, Shanshan Chen, Jing Peng, Xinyao Luo, Liya Wang, Ruoxi Liao, Yuliang Zhao, Shu Zhang, Baihai Su

**Affiliations:** 1 Department of Nephrology, Kidney Research Institute, West China Hospital, Sichuan University, Chengdu 610041, China; 2020224020032@stu.scu.edu.cn (Z.Z.); pengjing@stu.scu.edu.cn (J.P.); luoxinyao@stu.scu.edu.cn (X.L.); 2021324020024@stu.scu.edu.cn (L.W.); lrx-scu@scu.edu.cn (R.L.); zhaoyuliang@scu.edu.cn (Y.Z.); 2 Center of Gerontology and Geriatrics, West China Hospital, Sichuan University, Chengdu 610041, China; hxlijiameng@wchscu.edu.cn; 3 Department of Nephrology, The First Affiliated Hospital of Wenzhou Medical University, Wenzhou 325000, China; chenshanshan234@163.com; 4 Department of Nephrology, The Third People’s Hospital of Chengdu, Chengdu 610041, China; 5 Department of Emergency Medicine, West China Hospital, Sichuan University, Chengdu 610041, China

**Keywords:** acute kidney injury, NADPH oxidase 4, reactive oxygen species, endoplasmic reticulum stress

## Abstract

Rhabdomyolysis is a severe condition that commonly leads to acute kidney injury (AKI), with limited targeted treatments for rhabdomyolysis-induced AKI (RIAKI) adding to the challenge. Emerging evidence implicates nicotinamide adenine dinucleotide phosphate (NADPH) oxidase 4 (NOX4) in the pathological processes of various kidney diseases, but its role in RIAKI remains unclear. We applied renal tubular epithelial cell (RTEC)-specific NOX4 knockout and the NOX4 inhibitor GKT137831 to treat RIAKI in vivo and in vitro. We found that genetic and pharmacological inhibition of NOX4 protected against glycerol-induced renal dysfunction, mitigated inflammatory responses and attenuated apoptotic rates. Additionally, NOX4 blockade suppressed the accumulation of reactive oxygen species (ROS) and malondialdehyde (MDA), and enhanced the activities of antioxidant enzymes. Furthermore, NOX4 inhibition reduced the expression of endoplasmic reticulum stress (ERS)-associated proteins at both the RNA and protein levels. Collectively, these findings demonstrate that genetic and pharmacological suppression of NOX4 protects against RIAKI by reducing ROS generation, boosting antioxidant defense and inhibiting ERS activation. NOX4 inhibition may offer a potential approach for developing new treatment options for RIAKI.

## 1. Introduction

Acute kidney injury (AKI), termed rhabdomyolysis-induced acute kidney injury (RIAKI), is the most common and potentially life-threatening complication of rhabdomyolysis and significantly improves the mortality associated with this condition [1,2,3]. Generally, 13–50% of rhabdomyolysis cases lead to AKI [3,4,5], with a case fatality rate ranging from 20% to 50% and peaking at 59% in severe patients [2,6,7,8]. Nevertheless, specific therapies for RIAKI are lacking because of its complex pathogenesis [9,10]. Currently, the primary clinical treatment for RIAKI is symptomatic and supportive, with a focus on continuous renal replacement therapy, volume therapy and the correction of water, electrolyte, and acid-base balance disorders, including the administration of sodium bicarbonate [6,9,11,12].

The pathogenesis of RIAKI is multifactorial, involving oxidative stress, the inflammatory response, renal tubular obstruction, renal vascular constriction, and additional pathophysiological mechanisms [9,10]. The intracellular contents of skeletal muscle cells are released into the systemic circulation following rhabdomyolysis [6,13]. Myoglobin, which is freely filtered by glomeruli, is reabsorbed by renal tubular epithelial cells (RTECs) via endocytosis, leading to excessive generation of reactive oxygen species (ROS) and severe oxidative damage to the kidney [6,14]. Previous evidence has suggested that oxidative stress mediated by myoglobin plays a critical role in kidney injury induced by rhabdomyolysis through inflammation and apoptosis pathways [10,15].

Nicotinamide adenine dinucleotide phosphate (NADPH) oxidase 4 (NOX4) is a transmembrane protein that is predominantly expressed in the kidney, where it is largely responsible for the generation of intrarenal ROS [16,17]. Importantly, NOX4-derived ROS have multiple biological functions as second messengers, mediating the regulation of ion channel activity, oxygen sensing, gene expression, cell senescence, and apoptosis [16,18]. Although various ROS-generating enzymes undoubtedly contribute to renal function reduction, NOX4 has been demonstrated to be the primary source of superoxide generation in both the rabbit kidney cortex and medulla [19]. Increasing evidence has reported that NOX4 plays a crucial role in redox processes associated with various types of renal diseases, including diabetic nephropathy [20], hypertensive nephropathy [21], acute kidney injury [22,23,24] and renal cell carcinoma [25,26]. Currently, the role of NOX4 in the pathogenesis of diabetic nephropathy and related complications has been confirmed [27]. We also observed the upregulation of NOX4 in various AKI models associated with cisplatin, lipopolysaccharide, and ischemia–reperfusion injury [28,29,30]. Our previous study demonstrated that NOX4 overexpression exacerbates sepsis-induced AKI (S-AKI) and cisplatin-induced AKI by increasing the generation of ROS, thereby triggering mitochondrial dysfunction, inflammation and apoptosis [28,29]. However, there is no prior study investigated the function of NOX4 in AKI related to rhabdomyolysis syndrome.

In the present study, we established a glycerol-induced RIAKI mouse model and used ferrous myoglobin-stimulated mouse kidney tubular epithelium (TCMK-1) cells to explore the function and regulatory mechanism of NOX4. We demonstrated that RTEC-specific NOX4 knockout and pharmacological inhibition of NOX4 with GKT137831 ameliorated RIAKI, mitigated inflammation, and suppressed apoptosis. Additionally, NOX4 might modulate ROS accumulation in renal tissues and influence endoplasmic reticulum (ER) stress in RTECs. Our findings revealed that NOX4 could potentially serve as a therapeutic target for RIAKI.

## 2. Materials and Methods

### 2.1. Reagents

Glycerol (G8190) was purchased from Solarbio (Beijing, China). Myoglobin from equine skeletal muscle (M0630) and L-ascorbic acid (A5960) were acquired from Sigma Aldrich (St. Louis, MO, USA). GKT137831 (S7171) was obtained from Selleck Chemicals (Shanghai, China).

### 2.2. Animals

The animal experiments were approved by the Animal Ethics Committee of Sichuan University (Chengdu, China) (Approval No. 20220412001) and were administered according to the National Institutes of Health Guidelines for the Care and Use of Laboratory Animals. Male C57BL/6J mice and RTEC-specific conditional NOX4 knockout mice on the C57BL/6J background were purchased from GemPharmatech Co. Ltd. (Nanjing, China). The mice were kept in the Animal Experiment Center of West China Hospital, Sichuan University (Chengdu, China), maintained with standard food and water and housed in a pathogen-free, humidity- and temperature-controlled environment with a 12 h light/dark cycle. The precise methodology for generating RTEC-specific NOX4 knockout mice has been detailed in our prior study [29], with Figure S1 providing a visual representation of this process. The genotypes of both the NOX4^tecKO^ (Cdh16-Cre^+^ NOX4^fl/fl^)and NOX4^fl/fl^ (Cdh16-Cre^-^ NOX4^fl/fl^, NOX4^fl/fl^) mice were validated via PCR analysis and DNA agarose gel electrophoresis, employing specific primers, as outlined in Table S1.

### 2.3. Animal Experiments

The RIAKI model was induced in male C57BL/6J mice (8 weeks old, 20–25 g) with intramuscular injection of 50% glycerol (8 mL/kg) into each bilateral hind limb using a 1 mL syringe fitted with a 26-gauge needle, with half the total dose administered to each side. All procedures were performed under deep anesthesia, utilizing isoflurane inhalation, to ensure pain relief during glycerol injection. Water and food were withheld for 24 h before glycerol injection to increase the incidence of renal injury. The mice were randomly divided into three groups: the control, glycerol, and glycerol + NOX4 inhibitor GKT137831 groups. For the NOX4 inhibitor GKT137831 group, GKT137831 was dissolved in 2% DMSO, 2% Tween 80, 30% PEG300 and ddH_2_O in sequence, and the mice were orally administered GKT137831 solution at a dose of 60 mg/kg/d for 5 consecutive days prior to glycerol injection. Moreover, both the control group and the glycerol group were administered an equal volume of solvent. For the RTEC-specific conditional NOX4 knockout mice, NOX4^tecKO^ genotype mice were chosen for the subsequent experiment, while littermates carrying the NOX4^fl/fl^ transgene were used as controls. NOX4^fl/fl^ and NOX4^tecKO^ mice (male, 8 weeks old, 20–25 g) were randomly assigned to four groups: NOX4^fl/fl^ control or glycerol groups and NOX4^tecKO^ control or glycerol groups. After 24 h of modeling, the mice were sacrificed by an overdose of sodium pentobarbital via intraperitoneal injection, and their blood and kidney samples were collected and stored at −80 °C for further experiments.

### 2.4. Cell Culture and TCMK-1 Treatment

Mouse kidney tubular epithelial cells (TCMK-1, ATCC^®^ CCL-139^TM^) purchased from ATCC (Manassas, VA, USA) were cultured in MEM/EBSS medium (C11095500BT, Gibco, Grand Island, NY, USA) supplemented with 10% fetal bovine serum (FBS, 35-081-CV, Corning, NY, USA) and 1% penicillin-streptomycin (15140-122, Gibco) at 37 °C in an environment of 5% CO_2_, and 95% relative humidity. We used cells from passages 3 to 10 for cell experiments. TCMK-1 cells were stimulated with 200 μM ferrous myoglobin containing 2 mM ascorbic acid for 24 h to establish an in vitro RIAKI model. The cells, at 50–60% confluence, were starved in MEM/EBSS supplemented with 0.5% FBS for 24 h before exposure. For the NOX4 inhibitor group, TCMK-1 cells were pretreated with 10 μM GKT137831 for 40 min.

### 2.5. Immunohistochemistry (IHC)

The tissue sections were dewaxed, dehydrated and then washed with phosphate-buffered saline (PBS, pH 7.4, BL302A, Cytiva, Marlborough, MA, USA). Endogenous peroxidase was removed using 3% H_2_O_2_, followed by antigen retrieval with citrate. The kidney slices were then incubated overnight at 4 °C with anti-NOX4 primary antibody (14347-1-AP, Proteintech, Wuhan, China, 1:100). Following the PBS washings, the sections were incubated at room temperature for 30 min with biotinylated anti-rabbit IgG. Finally, A 3,3′-diaminobenzidine substrate kit (G1212, Servicebio, Wuhan, China) was used for visualization. Images were captured using a panoramic scanning system (3DHISTECH, Budapest, Hungary) at a magnification of ×200.

### 2.6. Immunofluorescence Staining

NOX4 expression in the cortex of mouse kidneys was detected according to the manufacturer’s instructions. Initially, 4 μm thick paraffin-embedded kidney sections were prepared and then subjected to routine immunofluorescence staining protocols. These sections were subsequently incubated with anti-NOX4 primary antibody (14347-1-AP, Proteintech, 1:100) overnight at 4 °C, then washed with PBS and incubated with secondary antibodies for 2 h. Subsequently, cell nuclei were stained with 4′,6-diamidino-2′-phenylindole (DAPI, G1012, Servicebio) for 20 min at room temperature in the dark. Images were captured using a panoramic scanning system (3DHISTECH, Budapest, Hungary) at a magnification of ×200. Mean fluorescence intensity of each image was analyzed with Image J software (v1.54, NIH, Bethesda, MD, USA).

### 2.7. Hematoxylin & Eosin (H&E) Staining and Histologic Scoring

Kidney tissues were soaked and fixed overnight with 4% paraformaldehyde and embedded in paraffin. The 4 μm paraffin-embedded kidney sections were prepared for hematoxylin–eosin (HE) staining. The stained sections were examined under a light microscopy at magnifications of ×200 and ×400. Tubular injury was assessed based on the percentage of injured renal tubules that exhibited features such as brush border loss, tubular dilation/flattening, tubular degeneration, tubular cast formation, and vacuolization. The degree of histological injury was scored on a scale from 0 to 4, where 0 represented no damage, and 1, 2, 3, and 4 corresponded to <25%, 26–50%, 51–75%, and >76% damaged renal tubules, respectively.

### 2.8. Renal Function Assessment

We collected blood samples from the mouse retro-orbital vein 24 h after glycerol stimulation. These samples were incubated at room temperature for 2 h, after which the serum was separated by centrifugation. Serum creatinine (SCr) and blood urea nitrogen (BUN) were then assessed following the manufacturer’s instructions.

### 2.9. Enzyme-Linked Immunosorbent Assay (ELISA)

The pro-inflammatory cytokine levels, such as tumor necrosis factor-α (TNF-α) and interleukin-6 (IL-6), in the serum and TCMK-1 cell supernatant were detected with ELISA kits (88-7324-88, 88-7064-88, Thermo Fisher Scientific, Waltham, MA, USA).

### 2.10. Biochemical Tests

The serum malondialdehyde (MDA) content was determined with the MDA assay kit (G4320, Servicebio) through the quantification of thiobarbituric acid-reactive substances generation, with absorbance taken at 532 nm. Tissue samples were homogenized in ice-cold PBS. Following centrifugation of the supernatant, the protein concentration was quantified using the bicinchoninic acid assay method (P0012, Beyotime, Beijing, China). The activities of antioxidant enzymes total-superoxide dismutase (T-SOD) and catalase (CAT) in renal tissues were detected using commercial assay kits according to the kit’s instructions (G4306, G4307, Servicebio). The activities of both T-SOD and CAT were presented in enzyme units per milligram of protein (U/mg prot), where one unit (U) represented the amount of enzyme required to catalyze one unit of substrate specified for each assay kit.

### 2.11. Terminal Deoxynucleotidyl Transferase-Mediated dUTP Nick End Labeling (TUNEL) Staining

Apoptosis in renal tissues was identified by a TUNEL assay with an In Situ Cell Death Detection Kit (11684795910, Roche Life Science). DAPI was used for nuclear staining. Positive staining was detected by fluorescence microscopy (Carl Zeiss, Jena, Germany) at a magnification of ×200. For each sample, 10 areas were chosen at random to count the number of apoptotic cell nuclei, and these counts were averaged for statistical analysis.

### 2.12. Transmission Electron Microscopy

Briefly, fresh kidney cortices, approximately 1 mm^3^, were prefixed with 3% glutaraldehyde for 2 h at 4 °C. Then, all the samples were postfixed in 1% osmium tetroxide, dehydrated in a series of acetone, infiltrated in Epox 812 for a longer period, and embedded. The semithin sections were stained with methylene blue, and ultrathin sections were cut with a diamond knife and stained with uranyl acetate and lead citrate. The sections were observed by a JEM-1400-FLASH Transmission Electron Microscope (JEOL Ltd., Tokyo, Japan). Digital electron micrographs were randomly taken at ×8000 and ×30k magnification for each group in this study.

### 2.13. Cell Viability Assay

A Cell Counting Kit-8 (CCK-8, HY-K0301, MedChemExpress, Monmouth Junction, NJ,USA) was used to evaluate the cytotoxic effects of ferrous myoglobin and GKT137831 on TCMK-1 cells. According to the instructions, TCMK-1 cells were seeded in a 96-well plate (8000 cells/well) and incubated with several concentrations of ferrous myoglobin (0, 50, 100, 200, and 400 μM) or/and GKT137831 (0, 2, 5, 10, 20, and 40 μM) for 24 h. Afterwards, 10 μL/well CCK-8 solution was added to the cells in a 96-well plate for 1 h at 37 °C. The absorbance was measured at 450 nm using a microplate reader (Synergy Mx, Biotek, Winooski, VT, USA). Each experiment was repeated three times. Cell survival rate (%) = (OD^Treatment Group^ − OD^Blank Group^)/(OD^Control Group^ − OD^Blank Group^).

### 2.14. Calcein-AM/PI Double Staining Assay

The cell apoptosis of TCMK-1 cells was visualized through a Calcein-AM/PI Double Staining Kit (C542, DOJINDO, Kumamoto, Japan). TCMK-1 cells were plated at a density of 2 × 10^4^ cells/well in 24-well plates and incubated overnight. Next, the cells were subjected to treatment with either ferrous myoglobin or GKT137831 for a duration of 24 h. Then the cells were rinsed and incubated in 1 μΜ of Calcein-AM and 4 μM of PI in the dark for 20 min at 37 °C. The cells were stained with Calcein-AM and PI to differentiate between live and dead cells, and then visualized under a fluorescence microscope (Carl Zeiss, Jena, Germany). The ratio of live to dead cells in each image was analyzed with Image J software.

### 2.15. Annexin V-FITC/PI Assay

The apoptotic rate of cultured TCMK-1 cells was determined using an Annexin V-FITC/PI kit (FXP018Pro, 4A Biotech, Beijing, China). We collected both adherent cells and suspended cells in the supernatant according to standard instructions from the manufacturer, after which the cells were washed with cold PBS and resuspended in a buffer. Subsequently, 3 μL of Annexin V-FITC and 7 μL of PI were added to the cell suspension, and the samples were incubated in the dark at 37 °C for 10 min. Apoptotic cells were immediately detected by flow cytometry (Beckman Coulter, Brea, CA, USA).

### 2.16. ROS Detection

For ROS measurements, frozen renal sections (4 μm) were stained with 10 μM dihydroethidium (D7008, DHE, Sigma‒Aldrich) in a light-protected humidified chamber at 37 °C for 30 min. DHE primarily binds to superoxide anions (O_2_^−^) and emits red fluorescence upon oxidation. Nuclei were counterstained with DAPI. we subsequently observed the stained samples by fluorescence microscopy (Nikon, Tokyo, Japan). Mean fluorescence intensity of each image was analyzed with Image J software. Moreover, intracellular ROS levels in TCMK-1 cells were measured by flow cytometry (Beckman Coulter, Brea, CA, USA) following a 20 min incubation with 2′,7′-dichlorofluorescein diacetate (S0033, DCFH-DA, Beyotime) at a 1:1000 dilution at 37 °C according to the manufacturer’s guidelines.

### 2.17. Quantitative Real-Time Polymerase Chain Reaction (RT-qPCR) Analysis

Total RNA was isolated from renal tissues or TCMK-1 cells with a FastPure^®^ Cell/Tissue Total RNA Isolation Kit V2 (RC112-01, Vazyme, Nanjing, China). Complementary DNA was synthesized using HiScript II Q Select RT SuperMix for qPCR (R223-01, Vazyme). This was followed by real-time PCR using SYBR Green Supermix (Q411-02, Vazyme). In the context of qRT-PCR analysis, each target gene was compared with GAPDH as the internal reference control, and the 2^−∆∆Ct^ method was used for statistical analysis. The primers, which were synthesized by Tsingke Biotechnology (Beijing, China), are listed in Table S2.

### 2.18. Western Blot Analysis

Total proteins from kidney tissues or TCMK-1 cells were extracted using radioimmune precipitation assay lysis buffer (RIPA, P0013B, Beyotime Biotechnology). The proteins were separated by 8–12% SDS–polyacrylamide gel electrophoresis, transferred onto polyvinylidene difluoride (PVDF, IPFL00010, Millipore, Billerica, MA, USA) membranes, blocked for 1 h with 5% nonfat milk in Tris-buffered saline containing 0.1% Tween 20 (TBST) at room temperature, and incubated with the corresponding primary antibodies at 4 °C overnight. Then the membranes were further incubated with HRP-conjugated secondary antibodies at room temperature for 1 h. Finally, the membranes were treated with chemiluminescence reagents and the signals were imaged by a chemiluminescence imaging system (Bio-Rad Laboratories, Inc., Pleasanton, CA, USA). The grayscale of the bands was evaluated using Image J software to visualize the expression levels of these target proteins, with GAPDH serving as the internal loading control. The antibodies were listed in Table S3.

### 2.19. Statistical Analysis

Each experiment was repeated at least three times. All the data are expressed as the means ± standard deviation (SD) and analyzed using Prism software (GraphPad 9.0, San Diego, CA, USA). ANOVA, followed by Tukey’s post-tests or Dunnett’s T3 test, was used to determine the statistical differences among multiple groups. *p*-values < 0.05 were considered statistically significant.

## 3. Results

### 3.1. NOX4 Deficiency and GKT137831 Treatment Both Attenuated Glycerol-Induced RIAKI

**Figure 1:**
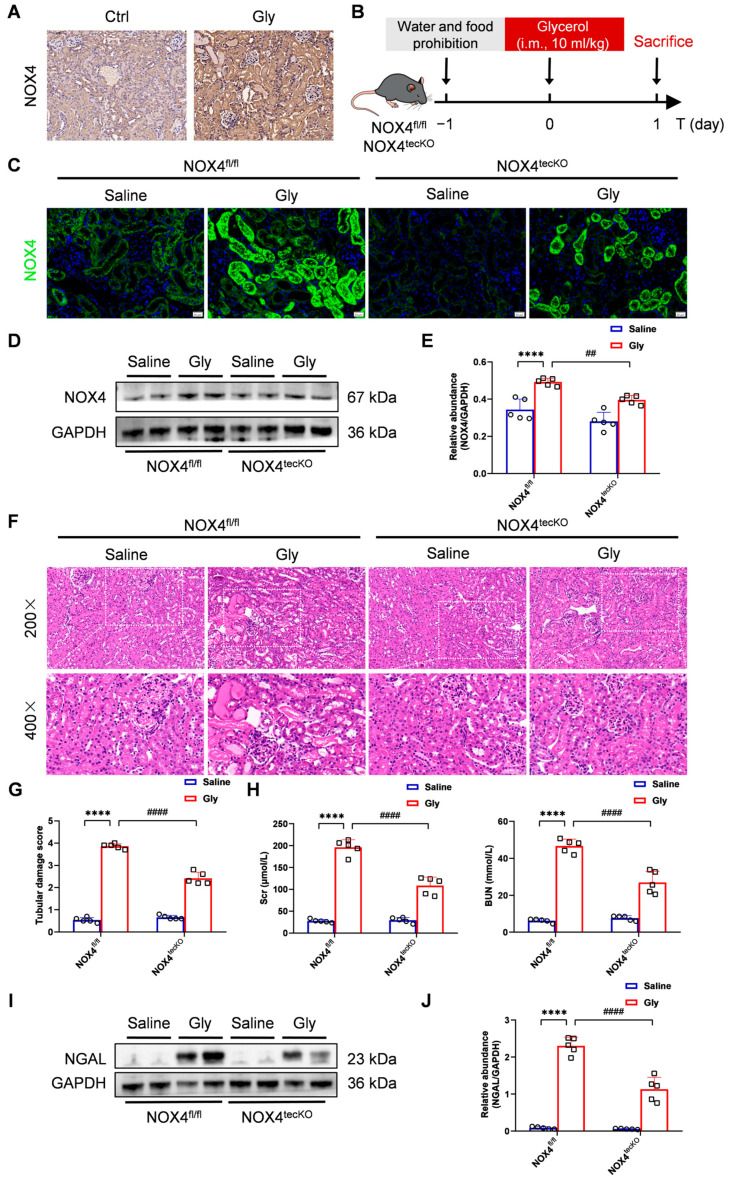
RTEC-specific deletion of NOX4 alleviated renal injury in glycerol-induced RIAKI mice. (**A**) Representative micrographs of immunohistochemical staining of NOX4 in the renal cortex (×400, scale bars = 20 μm). (**B**) Glycerol intervention in NOX4^tecKO^ and NOX4^fl/fl^ mice. (**C**) Representative micrographs of immunofluorescence staining of NOX4 (green) in the renal cortex (×400, scale bars = 20 μm). (**D**,**E**) Western blot analysis of NOX4 protein expression in the renal tissues and quantified by densitometry. (**F**,**G**) Representative images of HE staining (×200, scale bars = 50 μm; ×400, scale bars = 20 μm) and the tubular damage scores of kidney tissues. (**H**) Scr and BUN levels in different groups of mice. (**I**,**J**) Western blot analysis of NGAL protein expression in the renal tissues and quantified by densitometry. Data are represented as the mean ± SD, n = 5. **** *p* < 0.0001 vs. NOX4^fl/fl^/Saline; ^##^ *p* < 0.01, ^####^ *p* < 0.0001 vs. NOX4^fl/fl^/Gly. Gly: Glycerol.

To investigate the function of NOX4 in acute kidney injury (AKI), we established a mouse model of glycerol-induced AKI, which is a commonly used AKI animal model to imitate the pathological process of RIAKI in humans. We initially observed elevated NOX4 expression in the renal cortex of glycerol-induced AKI mice, as determined by immunohistochemical staining (Figure 1A). To evaluate the role of NOX4 in RTECs, we established the RTEC-specific conditional NOX4 knockout mice under the C57BL/6J background. The genotypes of NOX4^tecKO^ (Cdh16-Cre^+^ NOX4^fl/fl^) and NOX4^fl/fl^ (Cdh16-Cre^-^ NOX4^fl/fl^) mice were confirmed by DNA agarose gel electrophoresis (Figure S2). Although Cre recombinase does not achieve complete knockout of NOX4 in RTECs, this represents the technically feasible approach currently available for conditional gene targeting. Then, we induced RIAKI with glycerol in NOX4^tecKO^ and NOX4^fl/fl^ mice (Figure 1B). As illustrated in Figure 1C, NOX4 expression in the renal cortex (indicated by green fluorescence) was lower in the saline-treated group of NOX4^tecKO^ mice than in the NOX4^fl/fl^ littermates. In addition, RTEC-specific conditional NOX4 knockout hardly influenced the kidney histological structure or renal function of C57BL/6 mice (Figure 1F–H). After the injection of glycerol, NOX4 expression was upregulated in NOX4^fl/fl^ mice, but was significantly downregulated in NOX4^tecKO^ mice (Figure 1C). Moreover, Western blot revealed a significant reduction in the protein expression of NOX4 in the NOX4^tecKO^ mice (Figure 1D,E). These results shows that NOX4 was mostly inhibited in the RTECs of NOX4^tecKO^ mice.

Histological examination via H&E staining revealed that NOX4^fl/fl^ mice presented severe kidney pathological damage to the kidney following glycerol injection. There was less tubular injury, and lower tubular damage scores in NOX4^tecKO^ mice (Figure 1F,G). Compared with those in NOX4^fl/fl^ mice, serum creatinine and urea levels were apparently lower in NOX4^tecKO^ mice after 24h of glycerol administration (Figure 1H). Moreover, NOX4^tecKO^ mice effectively downregulated neutrophil gelatinase-associated lipocalin (NGAL), a well-established biomarker for AKI, by 58% (from 2.4-fold to 1.0-fold relative to GAPDH; *p* < 0.0001) compared with NOX4^fl/fl^ mice (Figure 1I,J). These findings collectively manifest that RTEC-specific conditional depletion of NOX4 attenuated glycerol-induced RIAKI in terms of histopathologic injury, renal function, or NGAL expression.

**Figure 2:**
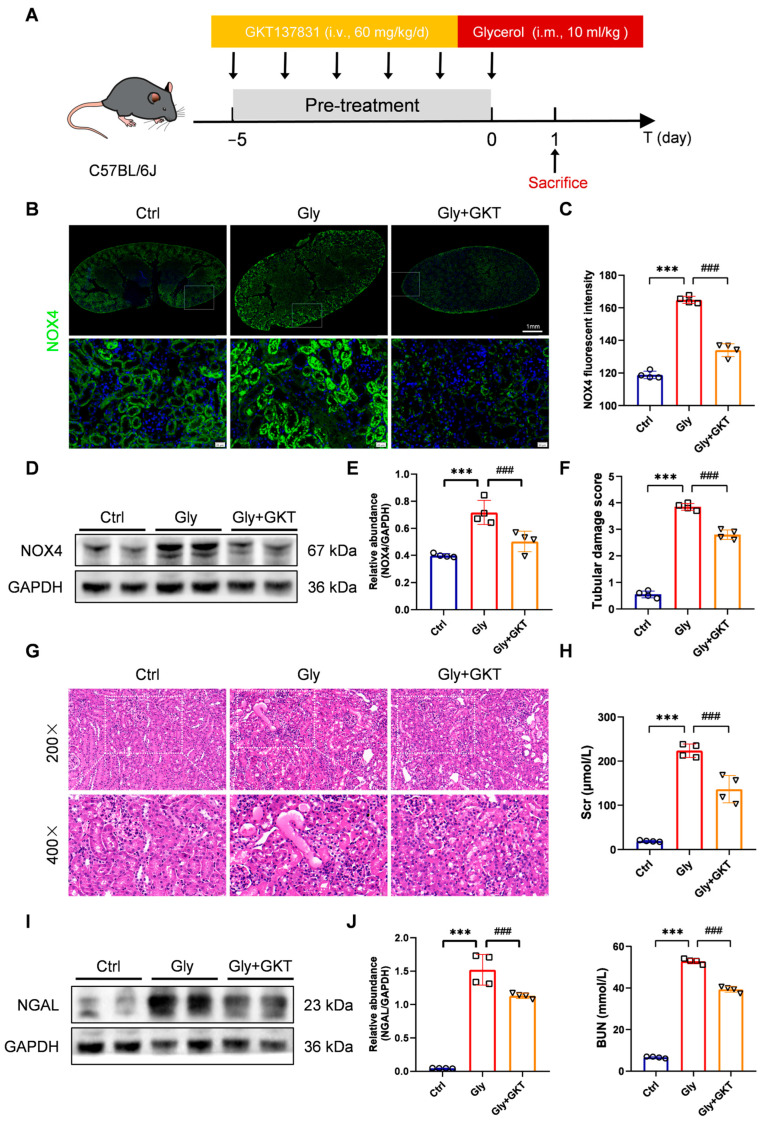
NOX4 inhibitor GKT137831 treatment ameliorated renal injury in glycerol-induced RIAKI mice. (**A**) GKT137831 intervention in a glycerol-induced RIAKI mouse model. (**B**,**C**) Overview and representative micrographs of immunofluorescence staining of NOX4 (green) in the renal cortex (×400, scale bars = 20 μm) and analysis of mean fluorescence intensity of NOX4. (**D**,**E**) Western blot analysis of NOX4 protein expression in the renal tissues and quantified by densitometry. (**F**,**G**) Representative images of HE staining (×200, scale bars = 50 μm; ×400, scale bars = 20 μm) and tubular damage scores of kidney tissues. (**H**) Scr and BUN levels in different groups of mice. (**I**,**J**) Western blot analysis of NGAL expression in the renal tissues and quantified by densitometry. Data are represented as the mean ± SD, n = 4. *** *p* < 0.001 vs. Ctrl; ^###^ *p* < 0.001 vs. Gly. Gly: Glycerol; GKT: GKT137831.

Then we examined the effect of Setanaxib (GKT137831), an effective small-molecule inhibitor of NOX4, on renal function and pathological changes in glycerol-induced AKI (Figure 2A). Immunofluorescence staining showed that GKT137831 decreased NOX4 fluorescence intensity in the renal cortex of glycerol-induced RIAKI mice (Figure 2B,C). Similarly, the expression of NOX4 in the kidneys was notably upregulated following glycerol injection, but mitigated in the presence of GKT137831 (Figure 2D,E). Overall, pretreatment with 60 mg/kg/d GKT137831 orally for five consecutive days effectively inhibited the expression of NOX4. A marked amelioration in histological kidney damage was observed in the GKT137831 group. (Figure 2F,G). GKT137831 administration decreased the increase in serum creatinine and urea levels in glycerol-induced RIAKI mice (Figure 2H). Similarly, Figure 2I,J demonstrated that GKT137831 treatment significantly reduced NGAL expression in glycerol-injured kidneys by 30% (from 1.59-fold to 1.11-fold relative to GAPDH; *p* < 0.01) compared with the glycerol group. These results suggest that GKT137831 protects against RIAKI via the inhibition of NOX4 expression.

Taken together, these findings indicated that glycerol induced NOX4 upregulation in RTECs, and that genetic and pharmacological inhibition of NOX4 protected renal function and attenuated pathological injury in glycerol-induced RIAKI mice.

### 3.2. NOX4 Inhibition Reduces Inflammation and Cell Apoptosis in Glycerol-Induced RIAKI Mice

**Figure 3:**
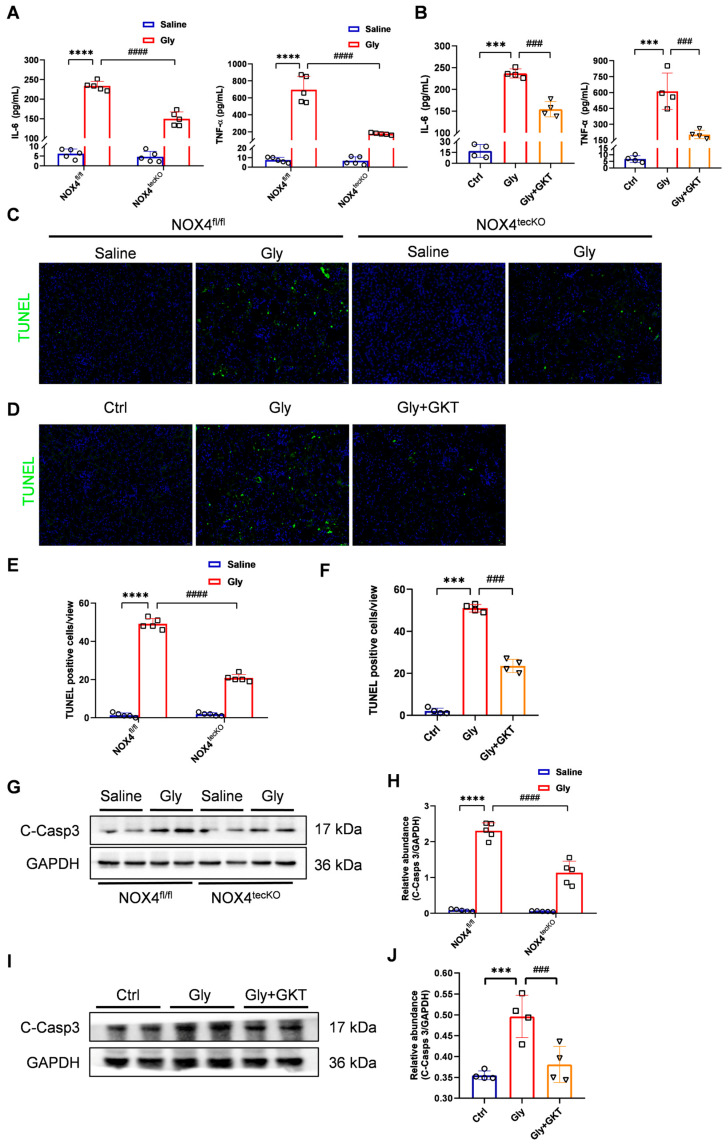
RTEC-specific knockout of NOX4 and NOX4 inhibitor GKT137831 treatment attenuated inflammation and apoptosis in glycerol-induced RIAKI mice. (**A**,**B**) Serum levels of IL-6 and TNF-α determined with ELISA kits. (**C**–**F**) Representative images of TUNEL staining (×400, scale bars = 20 μm) and quantification of TUNEL-positive cells in the kidney cortex. (**G**–**J**) Western blot analysis of C-Casp 3 expression in the renal tissues and quantified by densitometry. Data are represented as the mean ± SD, n = 5 or 4. *** *p* < 0.001, **** *p* < 0.0001 vs. Ctrl or NOX4^fl/fl^/Saline; ^###^ *p* < 0.001, ^####^ *p* < 0.0001 vs. Gly or NOX4^fl/fl^/Gly. Gly: Glycerol; GKT: GKT137831; C-Casp 3: cleaved-Caspase 3.

Recent studies have suggested that inflammation plays a crucial role in acute kidney disease, which is induced by various causes, such as ischemia and reperfusion injury, sepsis, cisplatin and rhabdomyolysis. To evaluate the effect of NOX4 suppression on systemic inflammation in RIAKI mice, we employed ELISA to measure the levels of inflammatory cytokines in the serum. Compared with those in the glycerol group, the serum levels of IL-6 and TNF-α were significantly lower in NOX4 deficiency or GKT137831-treated group (Figure 3A,B), indicating that NOX4 inhibition effectively mitigates inflammation in glycerol-induced RIAKI mice.

We further investigated the role of NOX4 in the apoptosis of RTECs in RIAKI mice. There was no difference in the TUNEL staining between NOX4^tecKO^ and NOX4^fl/fl^ mice in the saline group, but a reduction in TUNEL-positive cells was observed in NOX4^tecKO^ mice after glycerol injection, compared with NOX4^fl/fl^ mice (Figure 3C,E). As exhibited in Figure 3D,F, GKT137831 administration diminished the number of TUNEL-positive cells in the kidney of glycerol-induced RIAKI mice. Likewise, Western blot showed that the expression of cleaved-Caspase 3, a key apoptosis-executing protein, was evidently decreased with GKT137831 treatment and NOX4 deficiency (Figure 3G–J). Altogether, genetic and pharmacological inhibition of NOX4 both protected against apoptosis in glycerol-induced RIAKI mice.

### 3.3. NOX4 Inhibition Suppresses Oxidative Stress in Glycerol-Induced RIAKI Mice

**Figure 4:**
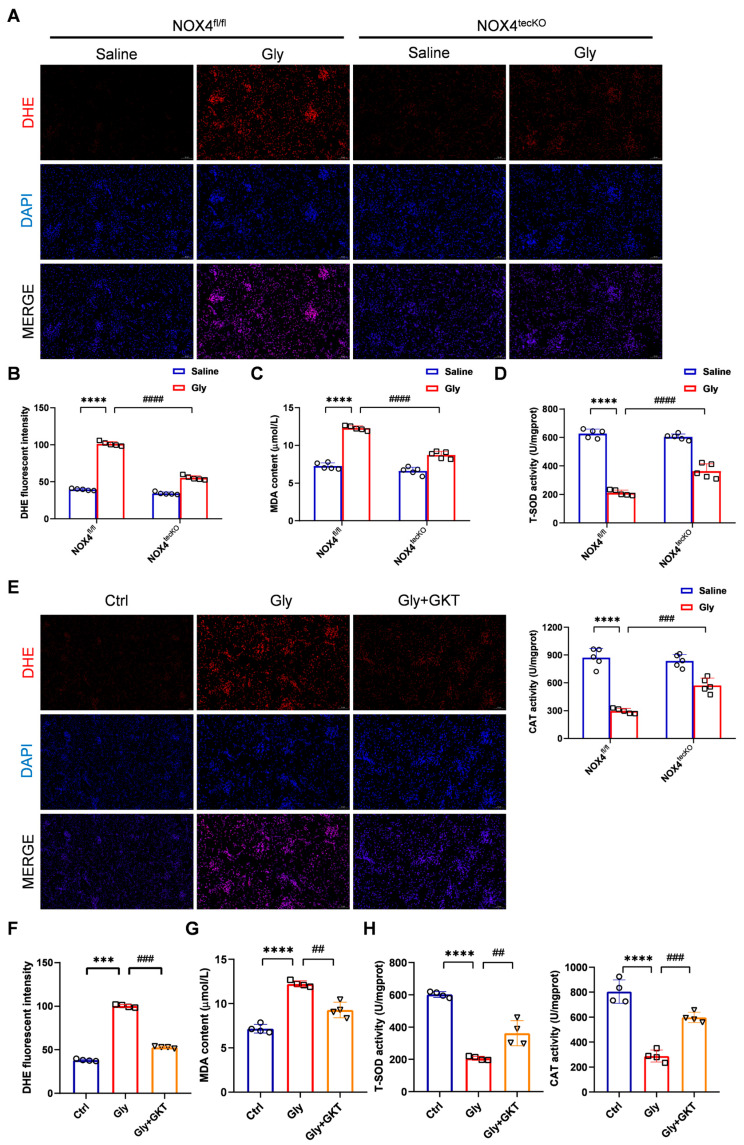
Genetic or pharmacological inhibition of NOX4 attenuated oxidative stress in glycerol-induced RIAKI mice. (**A**,**B**) Representative micrographs of DHE staining in renal tissues (×200, scale bars = 50 μm) and analysis of mean fluorescence intensity of DHE. (**C**) MDA levels in serum. (**D**) T-SOD and CAT enzyme activities in renal tissues. (**E**,**F**) Representative micrographs of DHE staining in renal tissues (×200, scale bars = 50 μm) and analysis of mean fluorescence intensity of DHE. (**G**) Serum MDA levels using MDA assay kit. (**H**) T-SOD and CAT enzyme activities in renal tissues. Data are represented as the mean ± SD, n = 5 or 4. *** *p* < 0.001, **** *p* < 0.0001 vs. Ctrl or NOX4^fl/fl^/Saline; ^##^ *p* < 0.01, ^###^ *p* < 0.001, ^####^ *p* < 0.0001 vs. Gly or NOX4^fl/fl^/Gly. Gly: Glycerol; GKT: GKT137831.

Many studies have shown that NOX4-derived ROS represent a key feature of a number of acute and chronic renal diseases. Thus, we marked superoxide anions (O_2_^−^) with a DHE probe to reflect the level of ROS in the kidney. Figure 4A,B illustrates an increase in the O_2_^−^ levels (indicated by red fluorescence) in glycerol-induced injured kidneys of NOX4^fl/fl^ mice, while NOX4^tecKO^ mice exhibited a reduction in average red fluorescence intensity, indicating attenuated ROS accumulation. Furthermore, we measured MDA levels and the activities of the antioxidant enzymes T-SOD and CAT in renal tissues. Compared with those in the saline group, serum MDA levels were distinctly higher in the glycerol group of NOX4^fl/fl^ mice, while RTEC-specific conditional NOX4 knockout reduced the MDA levels (Figure 4C). T-SOD and CAT activities were reduced after the glycerol induction in NOX4^fl/fl^ mice, but increased with genetic inhibition of NOX4 (Figure 4D). A similar tendency was also consistently observed in the GKT137831 group (Figure 4E–H). Hence, we suggested that NOX4 inhibition alleviated oxidative stress, including ROS and MDA accumulation, and promoted the antioxidative enzyme activities in renal tissues.

### 3.4. NOX4 Inhibition Suppresses Endoplasmic Reticulum Stress in Glycerol-Induced RIAKI Mice

**Figure 5:**
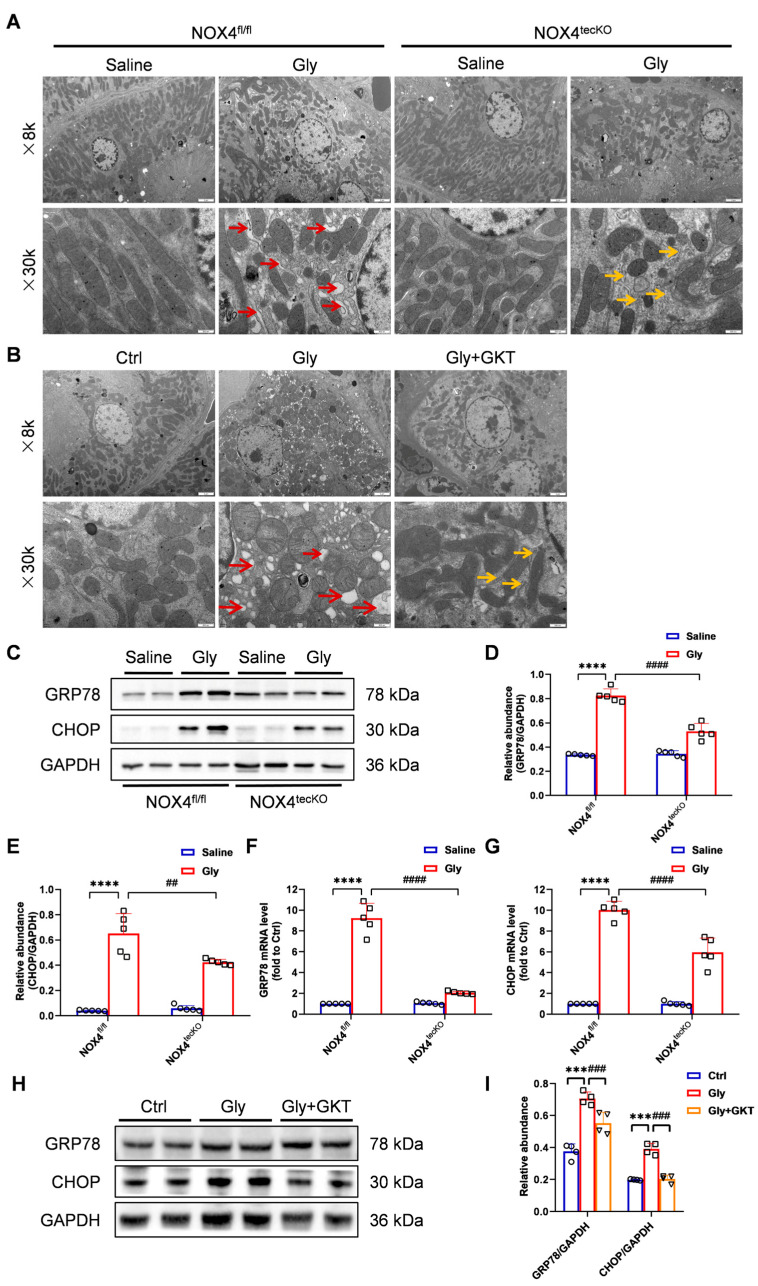
Genetic or pharmacological inhibition of NOX4 attenuated ERS signaling activation in glycerol-induced RIAKI mice. (**A**,**B**) Representative photomicrographs of ER in RTECs collected by transmission electron microscopy (×8000, scale bars = 2 μm; ×30,000, scale bars = 500 nm). The red arrow indicates damaged ER manifested as dilation and degranulation. The yellow arrow indicates recovered ER. (**C**–**E**) Western blot analysis of GRP78 and CHOP expression in renal tissues and quantified by densitometry. (**F**,**G**) Renal mRNA levels of GRP78 and CHOP measured by RT‒qPCR. (**H**,**I**) Western blot analysis of GRP78 and CHOP expression in renal tissues and quantified by densitometry. Data are represented as the mean ± SD, n = 5 or 4. *** *p* < 0.001, **** *p* < 0.0001 vs. Ctrl or NOX4^fl/fl^/Saline; ^##^ *p* < 0.01, ^###^ *p* < 0.001, ^####^ *p* < 0.0001 vs. Gly or NOX4^fl/fl^/Gly. Gly: Glycerol; GKT: GKT137831.

Diverse organelle dysfunctions play key roles in mediating inflammation and apoptosis in RIAKI. To further investigate the molecular mechanism by which NOX4 in RTECs affects RIAKI, we first observed the ultrastructural changes in the kidney cortex under the transmission electron microscopy. In the glycerol-induced RIAKI mice, the endoplasmic reticulum (ER) of RTECs exhibited severe defects, including enlargement, deformation, vesicle formation, together with the normal folded structures disappeared. However, both genetic and pharmacological inhibition of NOX4 reversed the aforementioned abnormal morphological alterations (Figure 5A,B). Based on the localization of NOX4 on the membrane of intracellular compartments such as the ER and mitochondria, we hypothesize that NOX4 inhibition might protect mice against glycerol-induced RIAKI by improving structural and functional changes in the ER, and mitigating the endoplasmic reticulum stress (ERS).

Accumulating evidence suggests that ERS plays a central regulatory role in RIAKI [31]. To determine the regulatory mechanisms underlying this process, we assessed the expression dynamics of key ERS-associated proteins, including the ER chaperone GRP78 and C/EBP homologous protein (CHOP), in a mouse model of RIAKI. CHOP is a major molecule regulating ER stress-induced apoptosis [32]. Compared with those in the control group, the mRNA and protein levels of the GRP78 and CHOP were evidently increased with glycerol induction, while RTEC-specific deletion of NOX4 reversed these abovementioned abnormalities (Figure 5C–G). Similarly, the protein levels of GRP78 and CHOP in injured kidneys were also downregulated by GKT137831 treatment (Figure 5H,I).

Collectively, NOX4 blockade significantly conferred renoprotection through the suppression of ROS accumulation and ERS signaling activation in vivo.

### 3.5. GKT137831 Suppressed NOX4 Expression in Ferrous Myoglobin-Stimulated TCMK-1 Cells

**Figure 6:**
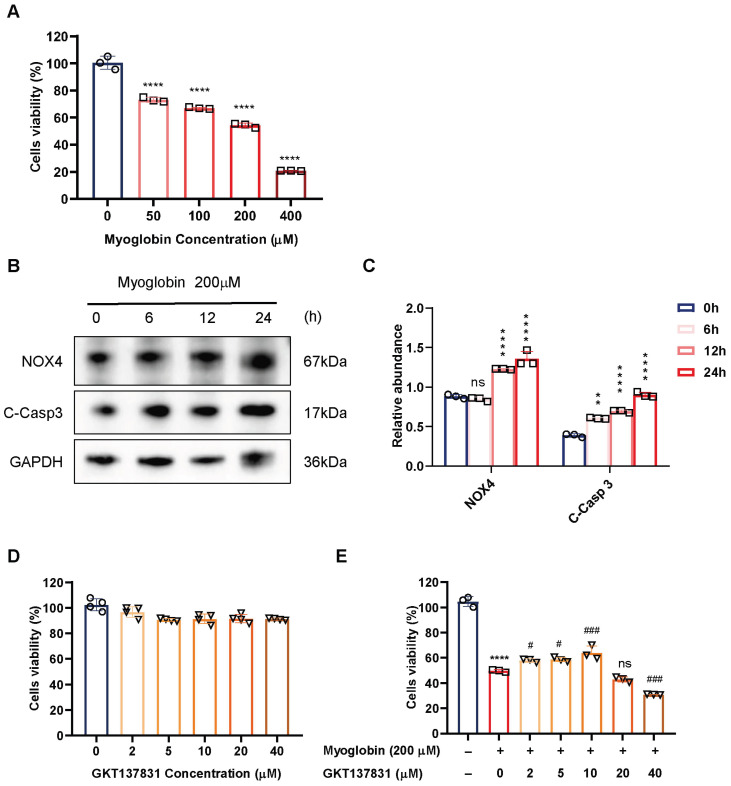
Effect of the dose and timing of ferrous myoglobin stimulation and GKT137831 treatment in TCMK-1 cells. (**A**) Cell viability of TCMK-1 cells treated with 0–400 μM ferrous myoglobin. (**B**,**C**) Western blot analysis of NOX4 and C-Casp 3 expression in TCMK-1 cells treated with 200 μM ferrous myoglobin for 0–24 h and quantified by densitometry. (**D**) Viability of TCMK-1 cells treated with 0–40 μM GKT137831. (**E**) TCMK-1 cells were pretreated with GKT137831 (0–40 μM) for 40 min and then exposed to ferrous myoglobin (200 μM) for 24 h. Viability of ferrous myoglobin-stimulated TCMK-1 cells treated with 0–40 μM GKT137831. Data are represented as the mean ± SD. n = 3. ns. no significant, ** *p* < 0.01, **** *p* < 0.0001 vs. myoglobin (0 μM) or myoglobin (200 μM) 0 h or myoglobin (200 μM)—with GKT(μM) -group; ^#^ *p* < 0.05, ^###^ *p* < 0.001 vs. myoglobin (200 μM) + with GKT(μM)—group. Mb: myoglobin; GKT: GKT137831; C-Casp 3: cleaved-Caspase 3.

Myoglobin is believed to be the main toxin causing the renal dysfunction in the pathophysiology of RIAKI [33]. According to the previous studies and the CCK-8 results, we selected 200 μM ferrous myoglobin to simulate damage to RTECs in the RIAKI model (Figure 6A). To determine whether myoglobin treatment triggers NOX4 upregulation, TCMK-1 cells were treated with 200 μM myoglobin for 0, 6, 12, and 24 h, and NOX4 protein levels were detected using Western blot analysis. As presented in Figure 6B,C, NOX4 protein levels significantly increased at 24 h following myoglobin treatment, and ferrous myoglobin upregulated the expression of NOX4 and cleaved-Caspase 3 in a time-dependent manner over 0–24 h. On the basis of these results, we stimulated TCMK-1 cells with 200 μM ferrous myoglobin for 24 h.

Then we conducted in vitro studies to examine the role of NOX4 protein in cellular responses triggered by myoglobin exposure in TCMK-1 cells. We first evaluated the nephrotoxicity of GKT137831 in vitro. The viability of TCMK-1 cells remained above 90% when the concentration of GKT137831 reached 40 μM, indicating minimal cytotoxicity (Figure 6D). To determine the optimum concentration of GKT137831 in vitro, a CCK-8 assay was employed to evaluate the cytoprotective effect of GKT137831 on TCMK-1 cells. We found that 10 μM GKT137831 significantly improved the viability of TCMK-1 cells treated with ferrous myoglobin (Figure 6E). As a result, we chose 10 μM GKT137831 as the optimal concentration for the subsequent experiments.

**Figure 7:**
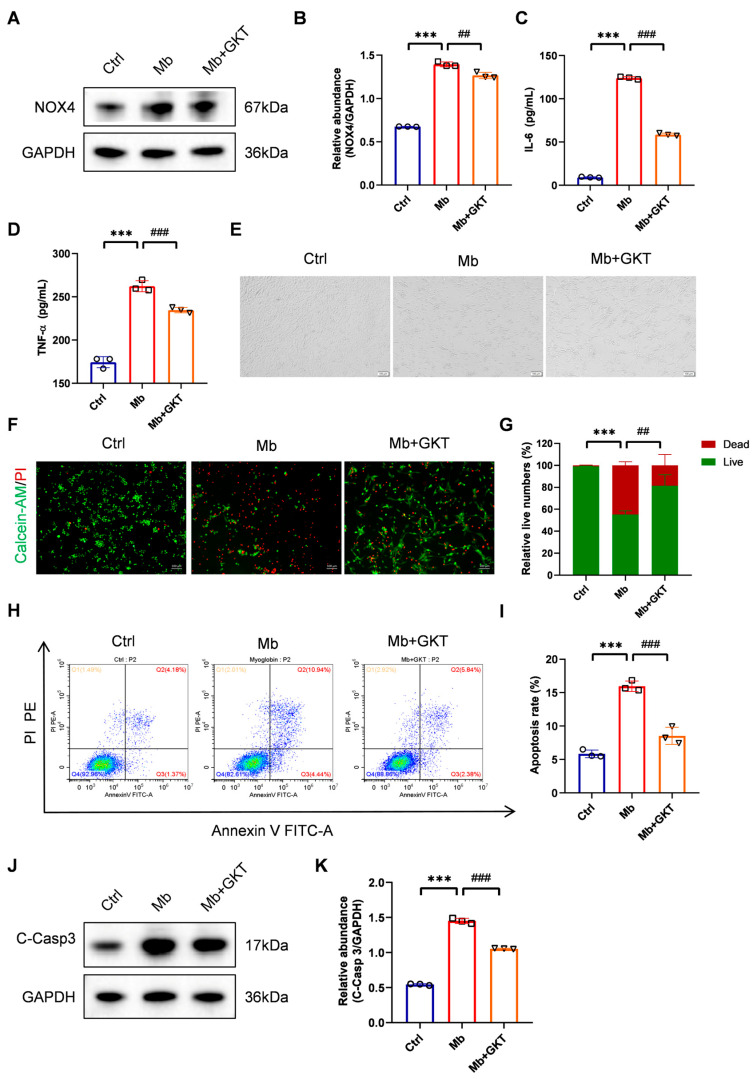
NOX4 inhibitor GKT137831 treatment suppressed inflammation and apoptosis in ferrous myoglobin-stimulated TCMK-1 cells. (**A**,**B**) Western blot analysis of NOX4 and quantification by densitometry. (**C**,**D**) The levels of IL-6 and TNF-α determined in the cell supernatant. (**E**) Representative images of TCMK-1 cells (×100, scale bars = 100 μm) under the optical microscope. (**F**,**G**) Live (green) and dead (red) TCMK-1 cells stained by Calcein-AM/PI staining (×100, scale bars = 100 μm) and the ratio of live to dead TCMK-1 cells. (**H**,**I**) Representative flow cytometric plots of TCMK-1 cell apoptosis and quantification of the apoptosis rate. (**J**,**K**) Western blot analysis of C-Casp 3 expression and quantified by densitometry. Data are represented as the mean ± SD. n = 3. *** *p* < 0.001 vs. Ctrl; ^##^ *p* < 0.01, ^###^ *p* < 0.001 vs. Mb group. Mb: myoglobin; GKT: GKT137831; C-Casp3: cleaved-Caspase 3.

As exhibited in Figure 7A,B, NOX4 expression was evidently upregulated in the ferrous-myoglobin group, whereas it was markedly reduced by GKT137831 pretreatment, indicating that GKT137831 suppressed NOX4 expression in the myoglobin-stimulated TCMK-1 cells.

### 3.6. GKT137831 Mitigates Inflammation and Cell Apoptosis in Ferrous Myoglobin-Stimulated TCMK-1 Cells

We further detected the anti-inflammatory and anti-apoptotic effects of GKT137831. Ferrous-myoglobin stimulation significantly increased the levels of TNF-α and IL-6 in the supernatant (*p* < 0.001), while pretreatment with GKT137831 effectively suppressed their secretion (Figure 7C,D, *p* < 0.001).

Under the light microscope, we observed that TCMK-1 cells became rounded and detached from the wall after ferrous-myoglobin stimulation, while GKT137831 treatment promoted more cells to adhere and grow (Figure 7E). We initially detected the cell apoptosis by a Calcein-AM/PI double staining assay. Figure 7F,G demonstrated that GKT137831 increased the proportion of live TCMK-1 cells after ferrous-myoglobin stimulation. Additionally, flow cytometry revealed that there was reduced apoptosis of TCMK-1 cells in the GKT137831 treatment group (Figure 7H,I). In accordance with the observed changes in the apoptosis ratio, treatment with ferrous myoglobin significantly upregulated cleaved Caspase-3 protein levels, while this effect was subsequently attenuated following incubation with GKT137831, indicating the inhibition of apoptotic signaling pathways (Figure 7J,K).

These findings offer additional support for the protective effects of GKT137831 against ferrous myoglobin-induced toxicity in TCMK-1 cells, such as inhibiting inflammation and apoptosis.

### 3.7. GKT137831 Reduced ROS Levels and Suppressed ERS in Ferrous Myoglobin-Stimulated TCMK-1 Cells

**Figure 8:**
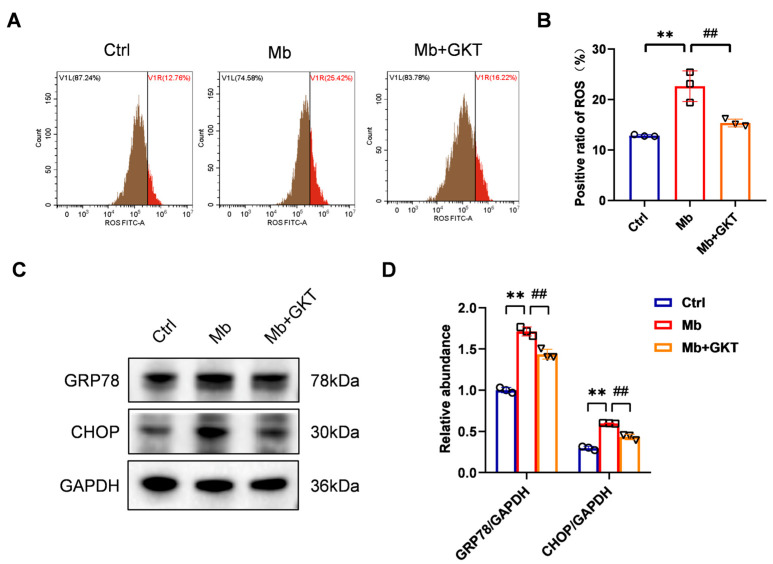
NOX4 inhibitor GKT137831 treatment suppressed ROS generation and ERS in ferrous myoglobin-stimulated TCMK-1 cells. (**A**,**B**) ROS levels in TCMK-1 cells were determined by flow cytometry. (**C**,**D**) Western blot analysis of GRP78 and CHOP expression and quantification with densitometry. Data are represented as the mean ± SD. n = 3. ** *p* < 0.01 vs. Ctrl; ^##^ *p* < 0.01 vs. Mb group. Mb: myoglobin; GKT: GKT137831.

We employed the DCFH-DA fluorescent probe to quantify the ROS levels in TCMK-1 cells. According to flow cytometry analysis, the level of ROS concentration in TCMK-1 cells was significantly greater after 24 h of myoglobin stimulation than that in the control group. This increase was effectively attenuated by specific inhibition of NOX4 (Figure 8A,B). Furthermore, to determine the regulatory effect of GKT137831 on the ERS level in ferrous-myoglobin-treated TCMK-1 cells, we systematically compared the expression profiles of key proteins across the experimental groups. Western blot analysis revealed that myoglobin exposure induced the upregulation of GRP78 and CHOP, which were markedly suppressed upon GKT137831 administration (Figure 8C,D). Collectively, these data establish that GKT137831 confers protective effects on TCMK-1 cells by modulating ROS generation and attenuating ERS-mediated apoptotic signaling.

## 4. Discussion

Herein, we report the role and regulatory mechanisms of NOX4 in the development of RIAKI both in vivo and in vitro. Genetic and pharmacological inhibition of NOX4 significantly preserved renal function and mitigated pathological damage in RIAKI mice by alleviating inflammation and apoptosis. Additionally, the protective role of NOX4 inhibition in renal injury might be attributed to attenuation of oxidative stress levels and regulation of excessive ERS. Our findings provide preliminary evidence supporting the potential renoprotective effects of NOX4 blockade in preclinical models of RIAKI, suggesting that this approach may warrant further investigation as a potential therapeutic target.

Rhabdomyolysis is a severe condition marked by extensive damage to skeletal muscles, leading to the release of byproducts from injured muscle cells into the bloodstream [6]. During rhabdomyolysis, the myoglobin released into the circulation is internalized by RTECs via endocytosis [34], where it triggers oxidative stress, inflammatory responses and cell cycle arrest within tubular cells, ultimately contributing to the development of AKI [35,36,37]. Most patients with AKI secondary to rhabdomyolysis clinically recover renal function, but RIAKI remains associated with increased mortality rates and a heightened risk of long-term progression to chronic kidney disease [38]. As the exact mechanism of RIAKI remains unclear, AKI patients are often treated with supportive therapy, such as continuous renal replacement therapy [9,39]. Therefore, a comprehensive elucidation of the underlying pathological mechanisms in RIAKI is imperative for the development of novel therapeutic strategies and potential pharmacological interventions.

The NOX family represents a preeminent source of ROS and exerts pivotal regulatory functions in redox signaling pathways [40]. NOX4, the most widely expressed isoform, is ubiquitously distributed in the kidney, including its vasculature, tubules, interstitium, and glomeruli, and is notably expressed in metabolically active renal tubular epithelial cells [17,41]. A previous study confirmed that NOX4 is responsible for ROS-induced fibroblast and mesangial cell activation and plays a vital role in kidney fibrosis [42]. Accumulating evidence further supports its involvement in AKI progression, including oxidative stress [43], inflammation [44], ferroptosis [45] and mitochondrial injury [29]. In cisplatin-induced AKI mice, NOX4-mediated superoxide formation increased proinflammatory cytokine production, both of which exacerbated cisplatin-induced renal damage [23,46]. A 2020 study revealed that the physical interaction between Smad3 and the NOX4 promoter region mediated ROS production and inflammation, promoting AKI susceptibility in diabetic mice [47]. Moreover, our prior studies revealed inherent NOX4 expression in mouse RTECs, while Maresin 1, an anti-inflammatory drug, protected against S-AKI by suppressing the NOX4/ROS/NF-κB pathway [28]. Additionally, genetic or pharmacological inhibition of NOX4 has been shown to exert protective effects against S-AKI through reducing ROS generation and NF-κB activation, thereby alleviating mitochondrial dysfunction, inflammation, and apoptosis [29].

Consistently, we found that NOX4 was also implicated in RIAKI pathogenesis, and NOX4 deficiency and GKT137831 treatment both attenuated RIAKI both in vivo and in vitro. Our research further contributed to the understanding of the role of NOX4 in rhabdomyolysis as an etiology of AKI, thereby highlighting the pivotal role of NOX4 in the pathogenesis of AKI. These findings collectively suggest that NOX4 may serve as a promising therapeutic target for mitigating oxidative stress injury in AKI.

To explore the functional role of NOX4 in rhabdomyolysis-induced AKI, we generated RTEC-specific conditional NOX4 knockout mice for mechanistic investigation. This advanced technology not only directly illustrates the function of NOX4 in RTECs but also effectively avoids potential non-renal functional abnormalities that might arise from the complete gene knockout [48,49]. Nevertheless, variations in Cre recombinase expression levels or inadequate cell type specificity are not uncommon [50]. Consequently, the knockout efficiency in conditional RTEC-specific NOX4 knockout models cannot achieve complete target gene disruption. Despite this limitation, this approach remains the optimal experimental strategy. In addition, we employed the NOX1/NOX4 inhibitor GKT137831 to suppress NOX4 activity specifically. NOX1 is highly expressed in the colon and plays a role in host defense and cell growth [51,52]. Although NOX1 can also be detected in renal cells, including glomerular endothelial cells, podocytes, and tubulointerstitial cells, the dominant NOX isoform in the kidney and the primary source of reactive oxygen species (ROS) in renal disease is widely recognized as NOX4 [53,54,55]. Hence, we propose that the renoprotective effects of GKT137831 against AKI are predominantly attributable to the selective inhibition of NOX4 enzymatic activity, rather than the involvement of NOX1.

ROS, generated through aerobic cellular respiration, participate in numerous cellular processes regulated by redox conditions. At the physiological level, ROS serve as critical signaling intermediates involved in key physiological pathways, including intracellular signal transduction, metabolism, immune and hypoxic responses, and transcriptional regulation [55,56]. Excessive or insufficient production of ROS destroys the balance between oxidation and antioxidants in cells. Therefore, as the main source of ROS in tissues and cells, NOX4 can act as a double-edged sword for the kidneys, resulting in conflicting results [57]. A particular research group reported that NOX4 plays a protective role against kidney interstitial fibrosis in obstructive kidney diseases by mitigating oxidative stress, promoting micro-vascularization, and inhibiting TGF-β-induced apoptosis [58]. The NOX4-regulated signaling cascade robustly promotes cellular viability and maintains translation initiation factor 2α phosphorylation, thereby mitigating ischemia–reperfusion induced AKI [59]. The available evidence indicates that the role of NOX4 markedly fluctuates depending on the specific type and stage of renal disease. Therefore, a systematic assessment of the potential unfavorable outcomes arising from NOX4 inhibition strategies within diverse clinical contexts of AKI is imperative.

The intracellular localization of NOX4 is still controversial. NOX4 is localized to multiple subcellular compartments, including the endoplasmic reticulum (ER), nucleus, plasma membrane, focal adhesions and mitochondrial membrane [18]. Karen Block provided evidence that functional NOX4 is present and regulated in mitochondria in the rat kidney cortex [60]. In vascular endothelial cells, both tagged and endogenous NOX4 strongly colocalize with the ER according to biochemical fractionation, live cell imaging, and immunoelectron microscopy results [61,62]. Moreover, accumulating evidence has elucidated the molecular mechanisms underlying the role of NOX4 in ER signaling.

Endoplasmic reticulum stress (ERS), characterized by the accumulation of misfolded client proteins within the ER lumen, triggers the unfolded protein response (UPR) to restore proteostasis by initiating three ER membrane sensors, including activating transcription factor 6 (ATF6), the inositol-requiring enzyme 1α (IRE1α) and PRKR-like endoplasmic reticulum kinase (PERK) [63,64]. During cellular stressors such as oxidative insults, the capacity of the ER to process properly folded mature proteins can be overwhelmed, resulting in the accumulation of misfolded or unfolded proteins [65]. Current evidence suggests that ERS is a key cellular stress condition that significantly exacerbates AKI and contributes to the progression of pathophysiological processes [63,65,66]. Prolonged activation of the PERK signaling pathway has been implicated in the induction of apoptosis, a process that is predominantly mediated by the C/EBP homologous protein (CHOP). As a major component of ER stress-induced apoptosis, CHOP upregulates pro-apoptotic genes while downregulating anti-apoptotic genes, ultimately resulting in programmed cell death [32]. Furthermore, eukaryotic initiation factor 2α (eIF2α) and X-box binding protein 1 (XBP1) serve as critical intermediates in the UPR signaling cascade, enhancing the activation of the NF-κB signaling pathway to promote pro-inflammatory IL-6 and IL-8 production [63,67]. In our study, we found that genetic and pharmacological inhibition of NOX4 effectively alleviated ERS in RTECs. This reduction in ERS was accompanied by decreased expression of CHOP, thereby attenuating both cellular inflammatory responses and apoptosis.

Crucially, NOX4-derived ROS generated within the ER serve as critical signaling intermediates, initiating autophagy by activating the EIF2AK3/PERK-EIF2S1/eIF2α-ATF4 pathway in cardiomyocytes under energy deprivation [68]. NOX4 also upregulates the classical ER stress response, including IRE1α sulfonation and the RIDD phenomenon, to exert its anticancer effects [69]. Existing studies have shown that NOX4 induces the UPR in the ER and leads to cardiomyocyte death through CHOP in ischemia–reperfusion (IR) myocardial injury [70]. Blunting NOX4 upregulation may alleviate diabetic nephropathy, partially by inhibiting ERS and inflammatory responses, thereby mitigating renal parenchymal damage [71]. The complex interplay between NOX4 and ERS-related signaling cascades operating through transcriptional regulatory circuits and epigenetic regulatory networks merits comprehensive exploration.

This study has a few limitations. First, the most significant limitation of this study is the prophylactic administration of the NOX4 inhibitor GKT137831 prior to glycerol or ferrous-myoglobin induction, which contrasts with clinical practice where AKI is an acute, unexpected event requiring intervention post-injury. Consequently, the intervention employed in this study—while valuable for mechanistic exploration—limited the direct translational relevance of these findings to patient treatment. Additionally, GKT137831 requires 3–10 days of continuous administration in animal models to achieve great NOX4 inhibition effects. However, our 24 h post-injury assessment window limits the evaluation of therapeutic efficacy. Second, while NOX4 inhibition exerted robust protective effects against RIAKI through ERS modulation, the precise genomic and transcriptional regulatory mechanisms underlying this protective pathway remain to be fully elucidated. Third, due to resource constraints, we were unable to employ cellular models for NOX4 gene silencing and overexpression studies, a limitation that will be addressed in subsequent studies. However, whether NOX4 blockade can reduce the risk of AKI in patients with rhabdomyolysis remains unknown. Nevertheless, our findings underscore the therapeutic potential of NOX4 as a novel molecular target for RIAKI, a hypothesis that warrants further investigation in both preclinical models and clinical trials.

## 5. Conclusions

In summary, our study revealed that NOX4 regulates inflammatory reactions and renal tubular injury. Additionally, NOX4 may induce severe oxidative stress and activate ERS both in vivo and in vitro. These observations establish NOX4 expression modulation as a promising therapeutic strategy for RIAKI management, offering new avenues for translational research in AKI prevention.

## Data Availability

All data generated or analyzed during this study are available from the corresponding author upon reasonable request.

## Supplementary Materials

The following supporting information can be downloaded at https://www.mdpi.com/article/10.3390/antiox14101162/s1. Supplementary Figures: Figure S1: Generation of renal tubular epithelial cell-specific (RTEC-specific) NOX4 knockout mice. Schematic of NOX4^flox/flox^ (NOX4^fl/fl^) mice generation by CRISPR/Cas9-stimulated homologous recombination and design strategy of RTEC-specific NOX4 knockout (NOX4^tecKO^) mice. Figure S2: Successful transmission of Cdh16-Cre and NOX4^fl/fl^ was confirmed by PCR genotyping. M marker; WT wild type. Supplementary Tables: Table S1: Primer sequences used in PCR assay of the genotype of NOX4^fl/fl^ mice (Cdh16-Cre-NOX4^fl/fl^) and NOX4^tecKO^ mice (Cdh16-Cre+ NOX4^fl/fl^). Table S2: Sequences of the primers for quantitative real-time PCR. Table S3: Antibodies used in Western blot.
