# Supplementary material for: Genetic and Pharmacological Inhibition of NOX4 Protects Against Rhabdomyolysis-Induced Acute Kidney Injury Through Suppression of Endoplasmic Reticulum Stress
Source: Antioxidants (Basel). 2025 Sep 25;14(10):1162. doi: 10.3390/antiox14101162 (PMC12561498; doi:10.3390/antiox14101162)
Supplement: Supplementary file 1 [file antioxidants-14-01162-s001.zip › antioxidants-3824579-Supplementary materials.pdf]

Supplementary Figures

Figure S1: Generation of renal tubular epithelial cell-specific (RTEC-specific) NOX4 knockout mice. Schematic of $NOX4^{flox/flox}$ ($NOX4^{fl/fl}$) mice generation by CRISPR/Cas9-stimulated homologous recombination and design strategy of RTEC-specific NOX4 knockout ($NOX4^{tecKO}$) mice.

Figure S2: Successful transmission of Cdh16-Cre and $NOX4^{fl/fl}$ was confirmed by PCR genotyping. M marker; WT wild type.

Supplementary Tables

Table S1: Primer sequences used in PCR assay of the genotype of $NOX4^{fl/fl}$ mice (Cdh16-Cre-$NOX4^{fl/fl}$) and $NOX4^{tecKO}$ mice (Cdh16-Cre+ $NOX4^{fl/fl}$).

Table S2: Sequences of the primers for quantitative real-time PCR.

Table S3: Antibodies used in Western blot.

Figure S1

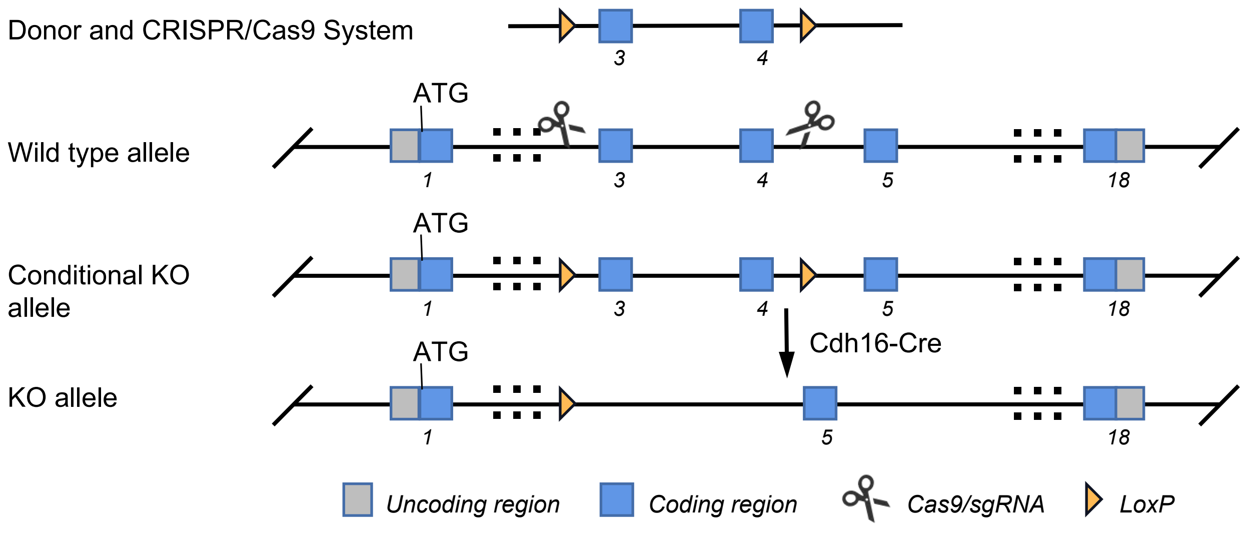

Figure S2

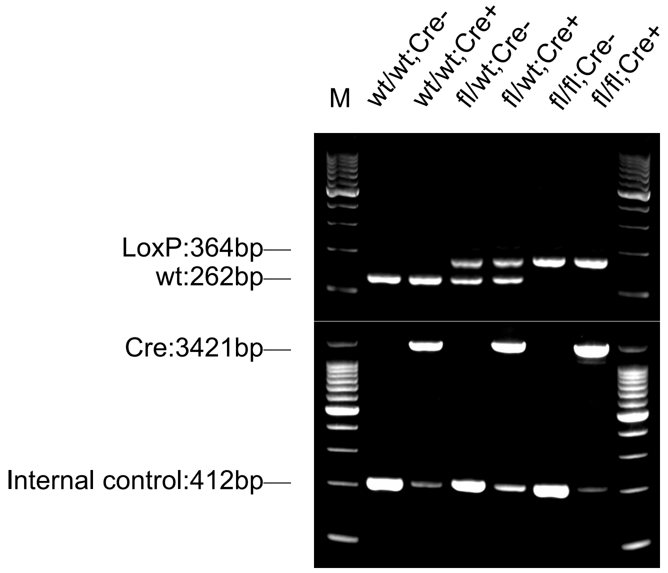

Marker: 200-4000bp

Table S1

Primer name	Sequence（5’-3’）	PCR Size	illustration
JS01996-Nox4-5wt-tF2	GATCAGGGAAACACTCAAGGGG	Flox: 364 bp	NOX4-LoxP
JS01996-Nox4-5wt-tR2	CCTAGATGGAAGATGCCTGAGATC	Wt: 262 bp	
H11-wt-tF1	CAGCAAAACCTGGCTGTGGATC	KI: 3421 bp	Cdh16-Cre
H11-wt-tR1	ATGAGCCACCATGTGGGTGTC	Wt:412 bp	

Table S2

Mouse gene	Sequence
F-GRP78	GTGTGTGAGACCAGAACCGT
R-GRP78	TAGGTGGTCCCCAAGTCGAT
F-CHOP	TCTTGAGCCTAACACGTCGATT
R-CHOP	ACGTGGACCAGGTTCTCTCT
F-GAPDH	AGGTCGGTGTGAACGGATTTG
R-GAPDH	TGTAGACCATGTAGTTGAGGTCA

Table S3

Primary antibodies	Dilution	Reference
anti-NOX4	1:2000	ab133303, Abcam
anti-NGAL	1:1000	ab23477, Abcam
anti-cleaved caspase-3	1:1000	9661, Cell Signaling Technology
anti-GRP78/BIP	1:5000	11587-1-AP, Proteintech
anti-GADD153/CHOP	1:1000	15204-1-AP, Proteintech
anti-GAPDH	1:5000	ab8245, Abcam
Secondary antibodies		
anti-mouse IgG H&L(HRP)	1:5000	SA00001-1, Proteintech
anti-rabbit IgG H&L(HRP)	1:5000	SA00001-2, Proteintech
anti-goat IgG H&L(HRP)	1:5000	SA00001-4, Proteintech
